# Residual Stresses Measurements in Laser Powder Bed Fusion Using Barkhausen Noise Analysis

**DOI:** 10.3390/ma15072676

**Published:** 2022-04-05

**Authors:** Alexandre Staub, Muriel Scherer, Pascal Zehnder, Adriaan Bernardus Spierings, Konrad Wegener

**Affiliations:** 1 Institute of Machine Tools and Manufacturing, ETH Zurich, 8092 Zurich, Switzerland; scheremu@student.ethz.ch (M.S.); pazehnde@ethz.ch (P.Z.); wegener@iwf.mavt.ethz.ch (K.W.); 2 Inspire, Innovation Center for Additive Manufacturing Switzerland (Icams), 9014 St Gallen, Switzerland; spierings@inspire.ethz.ch

**Keywords:** LPBF, residual stresses, Barkhausen noise, monitoring, MS300, 1.2709

## Abstract

In recent years, the advancement of technology brought the laser powder bed fusion process to its industrialisation step. Despite all the advancements in process repeatability and general quality control, many challenges remain unsolved due to the intrinsic difficulties of the process, notably the residual stresses issue. This work aimed to assess the usability of Barkhausen noise analysis (BNA) for the residual stress in situ monitoring of laser powder bed fusion on Maraging steel 300 (18Ni-300/1.2709). After measuring the evolution of grain size distribution over process parameter changes, two series of experiments were designed. First, a setup with an external force allows to validate the working principle of BNA on the chosen material processed using LPBF. The second experiment uses on-plates samples with different residual stress states. The results show a good stability in microstructure, a prerequisite for BNA. In addition, the external load setup acknowledges that signal variation correlates with the induced stress state. Finally, the on-plate measurement shows a similar signal variation to what has been observed in the literature for residual stress variation. It is shown that BNA is a suitable method for qualitative residual stresses variation monitoring developed during the LPBF process and underlines that BNA is a promising candidate as an in situ measurement method.

## 1. Introduction

Laser powder bed fusion (LPBF; according to ISO/ASTM 52900) allows for the manufacturing of products with a complex design, not achievable with conventional manufacturing. Examples are tooling parts with cooling channels or complex-shaped lightweight components for aerospace and space applications. As noted by Bourell et al. [1], Schmidt et al. [2] and Patterson et al. [3], the residual stresses remaining in the parts are a major bottleneck for further industrial acceptance of the technology. Leading to in-process deformation, build job interruption and critical part distortion after removal from the build plate, residual stresses are an inherent characteristic of the process due to the high-temperature gradient induced by the laser beam. Many works have been performed to characterise residual stresses induced by the LPBF process. Withers and Bhadeshia [4] defined nomenclature for residual stresses depending on their scale in the part. The vast majority of the works on residual stresses in the LPBF process are from type I, as these stresses are the most relevant in terms of part deformation. A fast and efficient method to measure residual stresses occurring after the LPBF process is the quantification of geometrical deformation. This destructive method is commonly realised using a beam curvature after cut-off from the build plate, as used by Safronov et al. [5], while Ghasri-Khouzani et al. [6] used the overall deformation of the disc. Very few contributions have reported on the in situ monitoring of residual stresses; the most advanced is proposed by Bartlett et al. [7], who used digital image correlation to establish stress maps for simple geometries such as discs.

Residual stresses can appear in all metallic materials from external solicitation during the manufacture or use of a metallic product. One nondestructive technique used to monitor stresses is Barkhausen noise analysis (BNA), as described by Ilker Yelbay et al. [8], as well as per Ju et al. [9]. Barkhausen noise is a phenomenon that appears during the magnetisation of a ferromagnetic material. The domain walls start to move—those with the same magnetic orientation as the external field grow, and all the others shrink. The domain walls do not move continuously but are held up by different obstacles: microstructural changes, as shown by Buttle et al. [10]; defects in microstructure (e.g., dislocations) identified by Santa-aho [11]; and stresses, as discussed by Buttle et al. [12]. During BNA, the sample is magnetised using a changing positive and negative current. For each magnetisation, three bursts can be identified.

BNA is already used in various applications such as grinding burn and residual stress detection for gears, as shown by Santa-aho [11], and surface analysis of thermally affected surfaces after machining and grinding, as presented by Rosipal et al. [13]. Ilker Yelbay et al. [8] and Ju et al. [9] showed that residual stress measurements are possible for welded components. Vaidhianathasamy and Shaw [14] noted the different voltages required to magnetise regions with different hardness levels, showing that soft regions are magnetised at lower applied voltages than harder regions. The detection depth for Barkhausen noise has also been studied, and Moorthy et al. [15] found that lower frequencies (~0.2 Hz) are estimated to penetrate up to 635 μm into En36 steel used for the manufacturing of gears. Higher frequencies (70 kHz) are estimated only to penetrate 76 μm deep into the material. However, in their study, these values differed significantly depending on other parameters that affect the Barkhausen noise signal. An important material characteristic affecting Barkhausen noise is the microstructure of the sample. The microstructure of the sample directly affects the shape of the signal output, as it will define the domain configuration in the crystals. Characteristics of the Barkhausen noise signal affected by the microstructure of the sample and applied magnetising field are amplitude, peak width and shape. However, conclusions from Grum et al. [16] showed that residual stresses or induced stresses have a significantly higher impact on the Barkhausen noise signal, as they showed residual stresses variations in different microstructures (induced by different quenching temperatures) of carbon steel 1.0503 (C45E). While de Oliveira et al. [17] underlined the lack of literature on the application of Barkhausen noise analysis in AM, they produced first results showing the correlation between the Barkhausen noise root main square signal and residual stresses measured by XRD on 1.2709. The authors concluded that the analysis of this magnetic phenomenon can be used as a nondestructive method for residual stress monitoring.

This study intends to advance the understanding of BNA as a potential candidate for in situ stress monitoring in LPBF by performing microstructural assessment as a prerequisite to BNA. By measuring residual stresses in various conditions and near in situ conditions, it shows the suitability of such techniques for qualitative measurement on larger LPBF parts subject to in-process stress accumulation.

## 2. Materials and Methods

The first prerequisite for BNA is the use of a ferromagnetic material. For the sake of this study and according to its common use in AM, 1.2709 (Maraging steel 300, gas atomized, Concept Laser GmbH, Lichenfels, Germany) was selected. Another common material in AM that could be a good candidate for this study would be 1.4542 steel (17-4PH, gas atomized, Carpenter Technology Corporation, Philadelphia, PA, USA). However, pre-study trials showed that the microstructure of this material is not stable enough for use in BNA over varying process parameters. In addition, 1.4542 is mostly used for lightweight applications and, consequently, smaller parts. On the contrary, 1.2709 is often used for larger parts with much more material volume, such as dies and molds, which are more prone to the buildup of residual stresses. In addition, Bajaj et al. [18], in a recent review, reported a mix of cellular and dendritic microstructures after solidification, which is a good indicator for a reasonably low anisotropy. Microstructure analysis was performed using a scanning electron microscope (SEM) FERA Tescan (TESCAN, Brno, Czech Republic) equipped with an electron backscatter diffraction (EBSD) sensor. Acquired data from SEM were handled in the OIM software (v8, EDAX, Mahwah, NJ, USA) using the following procedure: grain confidence index standardisation with a grain tolerance angle of 5° and a minimum grain to anti grain ratio of 2 and neighbour orientation correlation with a grain tolerance angle of 5°. Pixels with a confidence index lower than 0.1 were removed. Microstructure investigations were on 10 mm-sided cubes and over the xz plane (view from the side of the sample in the build direction). All samples presented similar grain size distributions individually, well-fitting a power function [19] in the form

(1) $$y=a\;x^{b}\;,$$

where *y* represents the fraction area and *x* the fitted grain diameter.

To compare different grain size distributions, a power function was fit to a data set composed of all the distributions to be compared. The indicator for the fit of the function to the data set (i.e., how comparable are all points of a data set composed of several grain size distributions) is the coefficient of determination of the power function [20],

(2) $$R^{2}=1-\frac{S_{res}}{S_{total}}\;\;,$$

where *S_res_* and *S_total_* represent the residual sum of squares of the distribution and sum of squares proportional to the variance of the distribution, respectively.

Barkhausen noise analysis was performed using a magnetising yoke and a pickup coil isolated from each other and arranged in a sensor head. An alternating current was used to create a changing magnetic field in the sample. The pickup coil will record the changes in magnetic flux in the sample. In general, the main data being analysed to evaluate residual stresses is the root mean square (RMS) of the signal, as proposed by Ding et al. [21]. The RMS indicates the voltage of the electric field resulting from the magnetic field.

For the BNA, Rollscan 350 (Stresstech Oy, Finland) was used, and four different sensors were considered for the sake of this study, as summarised in Table 1. They represent a variety of different pickup and receiving coil configurations, hence inducing different magnetic fields in the material. Note that the S1-14-13-10 and S1-14-13-02 sensors did not produce successful results in initial testing, as their positioning was not repeatable due to the too-small contact area. Both positionings on the same and tilting angles were difficult to repeat and led to significantly different results from one measurement to another. The S1.18-13-01 sensor, a so-called “general purpose” sensor, even if more adapted to the measurement context, was barely successful. The curved pole pieces and spring-loaded pickup coils theoretically enable the measurement of different kinds of surfaces and sample forms. However, in this case, the repeatability of the measurement was, again, not stable enough. This was due to the smaller gauge volume measured, which could be different from one location to another (even with very small position variations), as well as the roughness of the surface, which appeared to increase the positioning difficulty of the spring-loaded pickup coil.

The S1-16-13-01 sensor, a so-called “flat sensor”, was the most successful, as the gauge volume was relatively large, the stress state of the material was averaged in this volume and repeatability was increased. In addition, the positioning of the sensor with respect to the sample was much more stable and easier than with other sensors. However, some tilting and instability in the measurement might also be experienced due to the slight vibration induced by magnetisation during measurement. For the additional experiments in this study, only the flat sensor was considered, as the other sensors did not qualify for the repeatable measurement of the flat but relatively rough surfaces of the as-processed material. Nevertheless, it is worth mentioning that the S1-14-13-10 and S1-14-13-02 sensors are usually used in sweeping measurements, in which the sensor is swept over a very smooth surface (such as a camshaft or gear tooth). The measurement proposed here is a static point-by-point approach for better stability. However, further development of the in situ approach might allow for a continuous on-the-fly measurement over the surface (during recoating time, for instance). In that case, it appears to be worth trying such sensors again and reconsidering their suitability.

**Figure 1:**
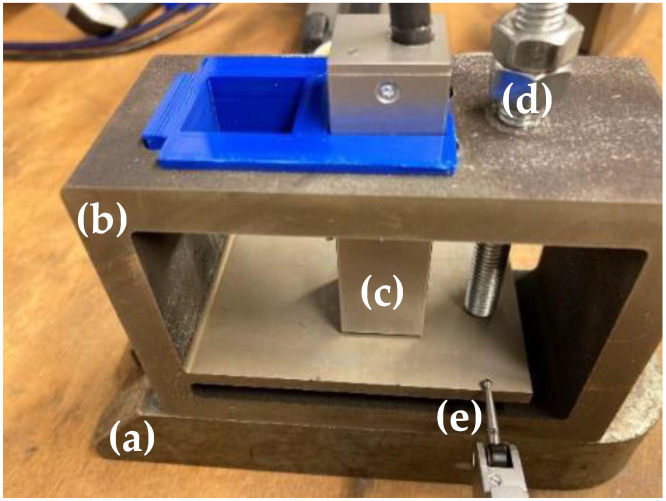
Bending beam method setup: (**a**) supporting plate; (**b**) setup manufactured with LPBF; (**c**) flat BNA sensor; (**d**) screw; (**e**) deflection measurement gauge.

To assess the suitability of BNA on the 1.2709 tool steel produced via LPBF (CL M2, Concept Laser GmbH, Lichtenfels, Germany), two experiments were conducted. In the first experimental setup, an external load was applied to measure the development of stresses in the material, as presented in Figure 1. As per the Euler–Bernoulli beam theory [22], for a beam of constant cross-section,

(3) $$M=-EI\frac{d^{4}\;w}{dx^{4}}\;\;\;,$$

where *w*(*x*) describes the deflection of the beam in the *z* direction at some position *x*. *M* is the bending moment of the beam, *E* is the elastic modulus and *I* is the second moment of area of the beam’s cross-section. Hence, stress on the top surface of the beam at a given location is linearly proportional to the force applied with the screw (Figure 1d) in the linear elastic domain considered here. In addition, for a given load, the stress at the surface increases from the tip to the built-in end. For this experiment, a setup with a single element composed of a beam of 80 × 50 × 5 mm with a constant section attached to a support frame was manufactured and stress relieved, as shown in Figure 1b. The support frame was equipped with a screw (Figure 1d) to apply a force on the tip of the beam. The deflection of the beam was measured at 10 mm from its tip with a micrometric gauge, as shown in Figure 1e. The whole setup was fabricated with a single piece of material using the LPBF setup described with the CBBB sample process parameters from Table 2 and stress annealed. The Barkhausen sensor was placed in contact with the beam at three different positions: 34, 41 and 46 mm from the tip. The measurement was continuously realised while bending the beam. This approach allows the sensitivity of BNA to be validated in a mechanically loaded component manufactured with LPBF. This first experiment is hereinafter referred to as the bending beam method.

Moreover, near in situ experiments were conducted to validate the BNA sensitivity to stress variations over different process parameters, as observed with XRD by Staub et al. [23]. For this experiment, samples of 40 × 40 × 10 mm^3^ were produced with parameters, leading to a material density of ρ > 99.5%. All samples were manufactured with a constant energy density of 47 J/mm^3^ (as per the definition of Röttger et al. [24]). Layer thickness and hatch distance were fixed at 50 and 90 µm, respectively. Laser power was varied in 50 W intervals from 150 to 350 W, while the scan speed was adjusted accordingly, as described in Table 2. The scanning strategy was a standard bidirectional strategy on the whole length of the sample with 90° rotation angle between successive layers. The samples for BNA were kept on the build plate after the LPBF process to avoid releasing of the residual stresses. The flat sensor was placed in the middle of the samples with the magnetic field parallel or perpendicular to the scan direction of the last layer. The measurements were repeated three times at the same location by two different operators to compensate for the very sensitive sensor position and tilt measurement method. This experiment is hereinafter referred to as the on-plate measurements.

## 3. Results

### 3.1. Microstructure Investigation

**Figure 2:**
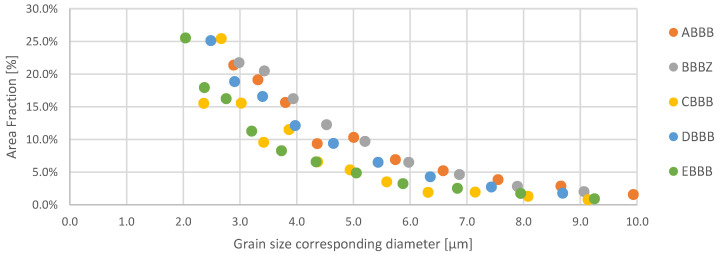
Grain size distribution of all samples together with a power fit function (dotted blue line, R^2^ = 0.879).

Overall, the grain size distributions showed good stability with variation in the laser power input in the plane considered. Although differences can be observed between the series, a power function fits the whole data set with an R^2^ of 0.88, indicating comparably stable grain size between samples, as shown in Figure 2. Hence, it can be considered that the relatively stable microstructures throughout the different samples considered in this study allow for the interpretation of changes in Barkhausen noise signal as changes in the stress states of the 1.2709 samples

### 3.2. Bending Beam Method

**Figure 3:**
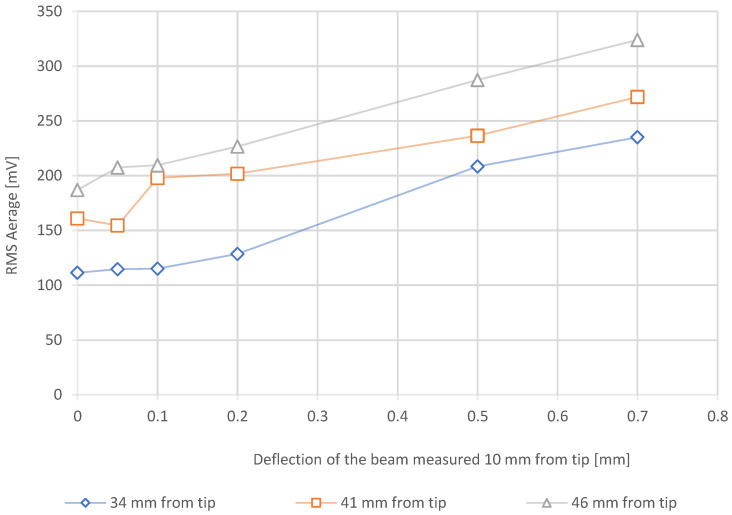
Barkhausen noise measurement with flat sensor on the deflection beam at various distances from the tip of the beam.

Figure 3 shows the linear response of the flat sensor to the application of a mechanical load on a simple beam. The linear behaviour of the Barkhausen noise RMS average signal is as expected because the beam theory predicts a linear increase in the stresses for a given beam position with a linear increase in applied force. The R^2^ coefficients for a linear fit are 0.97, 0.93 and 0.99 for the measurements at 34, 41 and 46 mm from the tip of the beam, respectively. In addition, for a known load, the sensor position also shows expected behaviour. Indeed, as the sensor becomes further from the tip of the beam, the signal increases along with stresses in the beam. However, these results also highlight the high sensitivity of the measurement technique, as the theoretical behaviour should be represented by three parallel lines with distances between each one proportional to the distance increase from the tip of the beam. However, the curves of the sensor response tend to be more stable with higher bending of the beam, suggesting that the measurement is more appropriate for relatively higher stress states or high variations. The bending beam method highlights that the LPBF-processed 1.2709 steel is a suitable material for the application of BNA to quantify the stress states of the material qualitatively.

### 3.3. On-Plate Measurements

**Figure 4:**
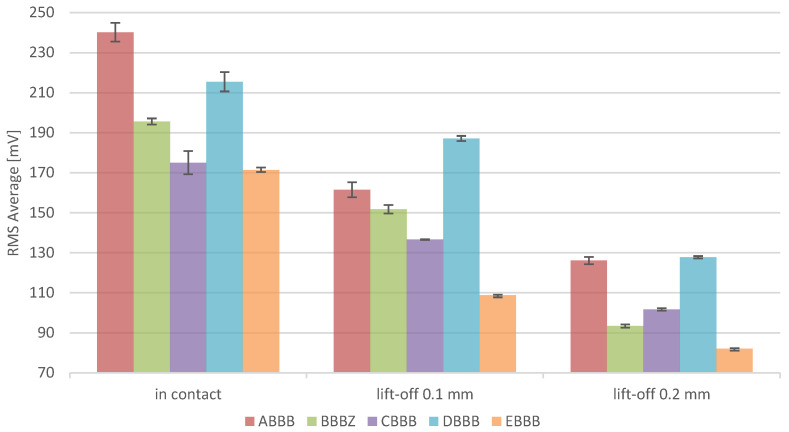
RMS average of different LPBF samples measured for in-contact measurements and lift-off measurements at 0.1 and 0.2 mm, where magnetic field is parallel to the last layer scanning direction.

The on-plate measurement shows particularly relevant results when the magnetic field is applied in the same direction as the scanning direction of the last layer. This is the direction where most stresses are present, as shown by Vrancken et al. [25] and Liu et al. [26]. The as-processed residual stresses of the parts, as presented in Figure 4, result in a decreasing Barkhausen noise RMS average from 150 W, reaching a minimum at 250 W. A further increase in the laser power leads to an increase in the residual stresses at 300 W, followed by a decrease of 350 W.

Although a different grade of steel was used in these experiments, these variations in the RMS average signal match the residual stress XRD observations for 1.4404 from Staub et al. [23]. The differences between the two observations are due to the differences in material and geometry, as both influence residual stresses. However, the general non-trivial behaviour (as linear behaviour would be expected, driven by a thermal gradient) of the stress conditions is present for both materials at higher laser powers.

In the case of a magnetic field perpendicular to the last scan track, the stress state is more complex and with lower magnitude versus the parallel case presented above, as discussed by Cheng et al. [27]. Consequently, BNA does not properly correlate with these local stress conditions. Hence, these data are not presented here. Additional measurements were also conducted on the edge of the samples, but the results could not be correlated to previously acquired stress data. The difficulty in interpreting the signals at these locations has two origins. On the one hand, the edge of the sample and, hence, the end or start of the scan track is not representative of the stress state in the core of the sample, as the melting conditions are not comparable. Either the melting is just starting and is not yet stable, or the front of the melt pool is solidified due to the discontinuity of the laser beam irradiation (end of scan track). On the other hand, the electromagnetic field is sensitive to edge effects and variation in the permeability of the material. Placing the sensor at the edge of a sample will generate a magnetic field that will encounter the edge of the sample, hence disturbing the measurement by passing from the metallic sample to the air.

The lift-off tests at 0.1 and 0.2 mm follow the same trend as identified for the in-contact measurements (Figure 3), showing that the implementation of such sensors in LPBF machines does not require direct contact with the material. On this occasion, it is clear that the measured media permeability is different compared with when it is in direct contact with the material, leading to a decrease in the signal amplitude. The magnitude of the drop can be influenced to some extent by increasing the magnetising voltage, which was realised in this case. These measurements are also in favour of measurements of relatively rough surfaces, as encountered in top surface LPBF samples, leading to different permeability as the air gap varies. This is also the case for the in-contact measurement of this data set, where a small air gap is also present, as the surface is not perfectly flat. Despite the more difficult measurement environment, the lift-off measurement showed a decrease of 46% and 73% of the standard deviation in the measurements for lift-off distances of 0.1 and 0.2 mm, respectively. This reduction is due to the high sensitivity of BNA to the sensor placement, particularly when contact with the sample can induce some tilting of the sensor. This tilting could originate from either the non-perfectly flat surface of the sensor or small vibrations due to the magnetisation currents. For the sake of the lift-off measurements, the sensor was attached to a micrometric axis, increasing the stability and repeatability of the measurements.

## 4. Discussion

By analysing grain size distribution in different processing conditions and conducting BNA in a mechanically induced stress state and an as-processed condition, this study allows to highlight that:

1. The LPBF manufactured sample complies with the necessary prerequisites of BNA, notably a good microstructure stability over process parameters variations.
2. BNA can successfully resolve stresses for 1.2709 steel in mechanically induced stress conditions. The increase in BNA signal follows a linear relation when stresses are applied linearly with confidence (R^2^) above 95%.
3. BNA shows promising results in the qualitative measurement of stresses in as-processed LPBF samples, comparable to XRD measurements presented in the literature. A stress accumulation or drift could be identified over the buildup of layers. Stress drift from 78% in the literature leads to a BNA signal drift of 82% in contact with the sample and 74% with a 0.1 mm lift-off.
4. BNA can be realised in a contactless manner with a recommended lift-off of 0.1 mm. This result advocates for its suitable use in rough LPBF parts as well as in situ contactless measurements.

These results underline the necessity for further research in the field of BNA applied to LPBF, particularly for the control of stress evolution during the build job. Indeed, although the measurement was not realised in the LPBF machine, only a few details are subject to change in comparison to the lift-off measurement in an in situ measurement setup. Notably, a temperature correction might be necessary. The opportunity for continuous measurement should also be studied for larger cross-sections. Further work on sensor design is also necessary to reduce the different uncertainties in the measurement presented.

## Figures and Tables

**Table 1:** Sensor section for Barkhausen noise analysis.

Sensor Designation	Sensor Picture	Magnetising Pole Length	Pickup Coil Length	Magnetising Pole Width	Pickup Coil Width	Contact Area Specifications
S1-16-13-01	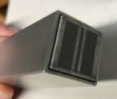	15 mm	15 mm	5 mm	1 mm	Flat contact area
S1-18-13-01	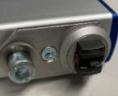	8 mm	3 mm	3.5 mm	1 mm	Spring-loaded pickup coil
S1-14-13-01	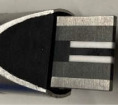	3 mm	1.5 mm	1 mm	1 mm	Flat contact area
S1-14-13-02	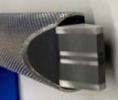	5 mm	1 mm	3 mm	3 mm	Flat contact area

**Table 2:** Sample identification and processing parameters.

Sample ID	Laser Power	Scan Speed	Energy Density
ABBB	150 W	709 mm/s	47 J/mm^3^
BBBZ	200 W	956 mm/s	46.5 J/mm^3^
CBBB	250 W	1182 mm/s	47 J/mm^3^
DBBB	300 W	1418 mm/s	47 J/mm^3^
EBBB	350 W	1655 mm/s	47 J/mm^3^

## Data Availability

Not applicable.
